# Beyond Chemodiversity: A General Biosynthetic Diversity Index for Plant Metabolic Architecture in Ecology, Evolutionary, and Bioprospection

**DOI:** 10.3390/molecules31122188

**Published:** 2026-06-22

**Authors:** Davyson de Lima Moreira, Renato Crespo Pereira, Ygor Jessé Ramos

**Affiliations:** 1 Research Institute of the Rio de Janeiro Botanical Garden, Rua Pacheco Leão 915, Jardim Botânico, Rio de Janeiro 22460-030, RJ, Brazil; 2 Department of Marine Biology, Universidade Federal Fluminense, Campus do Valonguinho, Outeiro de São João Batista, s/n, Centro, Niterói 24020-141, RJ, Brazil; rcrespo@id.uff.br; 3 Farmácia da Terra Laboratory, Faculty of Pharmacy, Federal University of Bahia, Rua Barão de Jeremoabo, 147, Ondina, Salvador 40170-115, BA, Brazil; ygor.jesse@ufba.br

**Keywords:** chemical diversity, biosynthetic pathways, metabolic architecture, general biosynthetic diversity index, plant secondary metabolism

## Abstract

Plant chemical diversity is commonly assessed using abundance-based metrics that treat metabolites as independent components, although these approaches do not explicitly represent how compounds are organized across biosynthetic routes. Here, we present a proof-of-concept application of the General Biosynthetic Diversity Index (GBDI), a pathway-informed descriptor of plant chemical mixtures that integrates relative metabolite abundance with biosynthetic-route attribution. The index was evaluated using hypothetical mixtures and essential oil datasets from *Piper rivinoides* Kunth (Piperaceae), including organ-level and ontogenetic comparisons. Limiting scenarios distinguished internally branched mono-pathway mixtures, balanced multi-pathway allocation, and ultra-dominated canalized profiles, supporting the use of GBDI as a descriptor of abundance-weighted biosynthetic architecture rather than pathway richness alone. In *P. rivinoides*, leaves showed the highest organ-level architectural diversity, branches showed focused monoterpene branching, and stems and roots showed more canalized arylpropanoid-rich architectures. Across ontogenetic stages, GBDI increased from juvenile to mature phases; however, this trend was interpreted descriptively because only five ordered phases were available. These findings position GBDI as a complementary metric for describing pathway-informed chemical organization and for generating testable ecological, evolutionary, and bioprospecting analyses.

## 1. Introduction

Chemical diversity in plants, particularly derived from specialized metabolites, constitutes one of the most remarkable features of plant biology and underpins a wide range of ecological and evolutionary processes. Specialized metabolites mediate interactions with herbivores, pathogens, pollinators, symbionts, abiotic stressors, and chemical communication processes, while also contributing to lineage diversification and environmental adaptation [1,2,3,4]. Owing to this multifunctional role, quantitative approaches capable of describing variation in plant chemical mixtures have become increasingly important in ecology, evolution, chemodiversity, chemophenetics, and bioprospecting [5,6].

Over the last two decades, chemical diversity indices have become particularly valuable for analyzing chemical phenotypic plasticity, chemotypes, and organ-specific allocation patterns, as well as for establishing connections between chemical phenotypes and ecological interactions [7,8,9]. In practical terms, chemical diversity indices provide a synthetic description of mixture complexity and dominance patterns, allowing researchers to compare mixtures through a single value or a small set of values that can be integrated into multivariate analyses, ecological gradients, or phylogenetic frameworks [8,10]. Most widely used chemodiversity indices consist of adaptations of metrics originally developed in community ecology [11,12,13,14,15,16]. Shannon-type indices quantify the uncertainty of randomly selecting a compound from a mixture; Simpson-type indices quantify dominance or concentration; and Pielou-type indices quantify evenness relative to an expected maximum value. These metrics are mathematically elegant and operationally simple. However, they share a conceptual assumption: individual metabolites are treated as independent units, analogous to species in a community. In many applications, this is convenient, but it is not always chemically or evolutionarily faithful to the way mixtures are produced [13,14,15,16]. Despite their broad application, these indices were not originally designed to represent the biosynthetic organization of plant chemical mixtures [13,14,15,16].

In these approaches, compounds differ only in abundance, and not in biosynthetic origin, although plant specialized metabolism is inherently hierarchical. Metabolites are generated in families through biosynthetic pathways that share precursors, enzymes, regulatory networks, and energetic demands. Therefore, two mixtures may present similar Shannon or Simpson values, even though they are produced by markedly distinct underlying metabolic architectures: one mixture may reflect deep diversification within a single pathway, whereas another may reflect shallow diversification distributed across multiple pathways. From a biosynthetic-evolutionary perspective, these two situations are not equivalent [12,17,18,19]. Thus, a methodological gap remains in the quantification of chemical diversity based on the logic of metabolic pathways.

Plant metabolites are produced by organized biosynthetic systems, including the acetate-mevalonate pathway, the methylerythritol phosphate pathway, the shikimate pathway, acetate-derived fatty acid pathways, and mixed-origin pathways. These routes differ in precursor use, enzymatic architecture, regulation, energetic investment, and ecological outcomes. A pathway-centered approach therefore evaluates not only which metabolites are present, but which routes are mobilized, how strongly they are expressed, and how internally diversified they are. In this framework, ecological or evolutionary interpretation remains hypothesis-generating unless supported by independent validation; nevertheless, a metric sensitive to biosynthetic architecture can reveal organizational patterns that are not captured by purely compositional indices [4,8,20,21,22,23,24,25,26].

This distinction suggests two complementary levels of chemical diversity. The first is compositional diversity, referring to the abundance distribution of metabolites within mixtures. The second is biosynthetic architectural diversity, referring to how metabolites are distributed among biosynthetic pathways and to the extent to which these pathways are internally diversified. Classical indices quantify the first dimension but do not satisfactorily account for the second. Importantly, biosynthetic architectural diversity should not be reduced to pathway richness alone, because metabolic diversification may emerge either from allocation among multiple routes or from extensive branching within a dominant biosynthetic pathway.

To address this gap, we present a proof-of-concept application of the General Biosynthetic Diversity Index (GBDI), a pathway-informed metric that integrates metabolite abundance with pathway-level organization. In operational terms, GBDI describes two linked components: relative allocation of abundance among biosynthetic routes and diversification of compounds within each route. The index is not proposed as a direct predictor of bioactivity, novelty, enzyme activity, or evolutionary mechanism; rather, it provides a quantitative descriptor that complements classical abundance-based chemodiversity indices.

Natural product discovery can benefit from descriptors that organize chemical information beyond compound counts, major peaks, or preliminary bioactivity. When integrated with metabolomics, dereplication, molecular networking, and taxonomically informed sampling, GBDI may add a pathway-level layer for prioritizing samples whose chemical investment is broadly distributed or internally branched. This application should be interpreted as a screening and hypothesis-generation tool, not as evidence that high GBDI necessarily predicts novelty or biological activity [26,27,28,29,30,31].

We hypothesize that mixtures that are chemically similar in richness or abundance may differ substantially in their biosynthetic architecture. We also assume that plant organs and developmental stages may exhibit different levels of biosynthetic diversification. Thus, the GBDI is expected to reveal patterns complementary to those obtained using classical diversity metrics. The genus *Piper* L. constitutes a relevant model for this type of approach due to its recognized chemical and ecological diversity. Species of this genus exhibit broad variation in the production of specialized metabolites, including volatile compounds of diverse biosynthetic origins. In this context, *Piper rivinoides* Kunth (Piperaceae) represents a suitable system for assessing biosynthetic variation across different organs and ontogenetic stages [19].

In this study, we present the mathematical formulation of GBDI, illustrate its behavior using hypothetical mixtures, and apply the index to the essential oils of *P. rivinoides* [19] to investigate spatial variation among plant organs and ontogenetic variation throughout developmental stages. We also interpret GBDI together with route-level decomposition, between-pathway diversity, intrapathway diversity, and dominance/canalization metrics, thereby separating compositional diversity, pathway allocation, and biosynthetic branching as complementary dimensions of plant chemical organization. In addition, we provide a supplementary comparison with Rao’s quadratic entropy, Hill-based diversity descriptors, and Bray-Curtis dissimilarity. This comparison is used to evaluate complementarity and potential redundancy descriptively, rather than to claim universal independence from existing metrics. By integrating abundance, within-pathway diversity, and distribution among biosynthetic routes, GBDI is presented as a complementary perspective for interpreting plant chemodiversity in ecological, evolutionary, and bioprospection.

## 2. Results and Discussion

### 2.1. General Rationale and Interpretative Framework of the General Biosynthetic Diversity Index

The GBDI was evaluated as a pathway-informed descriptor of plant chemical mixtures. Unlike conventional chemodiversity metrics, which generally treat metabolites as independent and exchangeable entities, GBDI explicitly couples the relative abundance of each metabolite with the total abundance allocated to the biosynthetic route from which that metabolite derives. The index therefore targets a hierarchical property of chemical organization: the distribution of metabolic investment across routes and the degree of diversification within those routes. The formal mathematical expression used to calculate the index is presented in Equation (1):

(1) $$GBDI=\ln\left(1+\sum_{i=1}^{n}(p_{i}\cdot P_{k}{)}^{\alpha }\right)$$

In this formulation, *p*_i_ is the relative abundance of metabolite *i*, *P_k_* is the cumulative relative abundance of the biosynthetic pathway *k* to which metabolite *i* is assigned, and alpha is a scaling parameter that controls the response of the index to dominance and diversification. In the main proof-of-concept analysis, alpha = 0.5 was used as the primary value because this concave scaling balances the influence of dominant constituents and minor but chemically relevant branches. To reduce arbitrariness, GBDI was also recalculated across an explicit alpha-grid (0.25, 0.50, 0.75, and 1.00), allowing the stability of organ-level and ontogenetic interpretations to be evaluated (Note S1; Table S1).

The logarithmic transformation compresses the pre-logarithmic aggregate while preserving the rank order among mixtures. This is useful because mixtures with many compounds or highly branched dominant routes can otherwise generate large, uncompressed scores. Consequently, GBDI should be interpreted as a relative and comparative measure of biosynthetic architecture, not as an absolute measurement of metabolic flux. The index is calculated from observed relative abundances and biosynthetic annotations; it does not directly estimate enzyme activities, transcript abundance, or true intracellular fluxes. The route-level decomposition used to identify the contribution of each biosynthetic class is presented in Equation (2) (Route-level contribution to the pre-logarithmic GBDI aggregate), whereas the proportional contribution of each route to the total pre-logarithmic aggregate is defined in Equation (3) (Percentage contribution of each biosynthetic route to the pre-logarithmic GBDI aggregate).

(2) $$C_{k}=\sum_{i\in k}(p_{i}P_{k}{)}^{\alpha }$$

(3) $$C_{k}(\%)=\frac{C_{k}}{\sum C_{k}}\times 100$$

The decomposition of GBDI into route-level contributions is essential for biological interpretation. A single final score can indicate that one mixture is more architecturally diversified than another, but it cannot, by itself, identify whether the difference arises from monoterpenes, sesquiterpenes, arylpropanoids, fatty-acid derivatives, or other route classes. Reporting $C_{k}$ and $C_{k}(\%)$ transforms the index from a scalar descriptor into an interpretable map of metabolic investment and clarifies whether high GBDI values reflect broad cross-pathway allocation, extensive intrapathway branching, or both. The complementary between-pathway and intrapathway Shannon descriptors used to support this interpretation are defined in Equations (4) (Shannon diversity among biosynthetic routes and effective-number transformation) and (5) (Intrapathway Shannon diversity and effective number of metabolites within route k).

For transparency and reproducibility, the Supplementary Material includes an automated spreadsheet for calculating GBDI and its companion analytical descriptors from pathway-attributed abundance matrices, including route-level contributions, between-pathway diversity, intrapathway diversity, compound- and pathway-level dominance, alpha-sensitivity outputs, null-model summaries, and a supplementary comparison with classical diversity and dissimilarity descriptors.

(4) Hpath=−∑k=1KPkln(Pk), D1path=exp(Hpath)

(5) qi∣k=piPk;Hwithin,k=−∑i∈kqi∣kln(qi∣k); D1within,k=exp(Hwithin,k)

where $p_{i}$ is the relative abundance of compound i, $P_{k}$ is the summed relative abundance of biosynthetic route k, and $q_{i|k}$ is the conditional relative abundance of compound i within route k. The term ^1^*D* corresponds to the Hill number of order 1, that is, the effective number of equally abundant categories.

GBDI is not expected to increase monotonically with the number of biosynthetic pathways. Its biological target is metabolic architecture. A mono-pathway mixture with extensive internal diversification can present a higher GBDI than a multi-pathway mixture in which several routes are present but weakly expressed or dominated by one compound. Therefore, GBDI should be read together with pathway richness, between-pathway diversity, intrapathway diversity, compound-level dominance, and pathway-level dominance. The conceptual matrix used to interpret GBDI values in relation to compound-level dominance ($p_{max}$), pathway-level dominance ($P_{max}$), biosynthetic interpretation, and architectural meaning is summarized in Table 1.

As shown in Table 1, low GBDI values should not be interpreted only as low richness; they may indicate compound-level canalization, pathway-level canalization, or both. Conversely, high GBDI values may arise either from distributed allocation across multiple biosynthetic routes or from strong intrapathway diversification within a dominant route. This distinction is central to the empirical interpretation of *P. rivinoides* because organs and ontogenetic stages (or phases) may occupy different positions within the same architectural framework.

### 2.2. Limiting Mixture Scenarios: Theoretical Behavior of GBDI

Three idealized scenarios were used to evaluate the behavior of GBDI under contrasting biosynthetic architectures: (1) a mono-pathway mixture with internal diversification, (2) a perfectly balanced multi-pathway mixture, and (3) an ultra-dominated multi-pathway mixture. These scenarios were not designed to reproduce a specific species but to test whether the metric discriminates between branching, balanced allocation, and canalization. The numerical structure of the limiting scenarios and their corresponding pre-logarithmic aggregates and GBDI values are summarized in Table 2 (see Supplementary Material).

In these hypothetical examples, the letters A and B are only arbitrary labels used to distinguish biosynthetic routes. The notation *P*A means the cumulative relative abundance assigned to route A, calculated by summing the relative abundances of all metabolites allocated to that route. Likewise, *P*B represents the cumulative relative abundance assigned to route B.

In the mono-pathway scenario, all four metabolites are assigned to the same hypothetical biosynthetic route, designated route A. Because their relative abundances sum to 1.00 (0.40 + 0.30 + 0.20 + 0.10), the total route-level abundance of route A (*P*A) is 1.00. Under this condition, the coupling term *p*_i_ × *P*_k_ becomes *p*_i_ × 1 and therefore reduces to *p*_i_. The index then behaves as a concave abundance aggregation within a single biosynthetic module. The resulting GBDI value (1.079) is higher than those of the two-pathway examples because the only route considered is internally branched into multiple relevant products. This result shows that biosynthetic diversification can occur within one route and that the number of routes alone is not the object measured by the index.

In the balanced multi-pathway scenario, two pathways contribute equally and the four metabolites are evenly distributed. Although the mixture contains more biosynthetic routes than the mono-pathway case, the pathway-level coupling term reduces the contribution of each metabolite because each pathway accounts for only half of the total mixture. The intermediate GBDI value (0.881) therefore reflects a portfolio-like allocation strategy rather than maximal architectural diversification. This behavior is biologically desirable: a mixture should not be considered highly biosynthetically diversified merely because it contains several weakly expressed routes.

**Figure 1:**
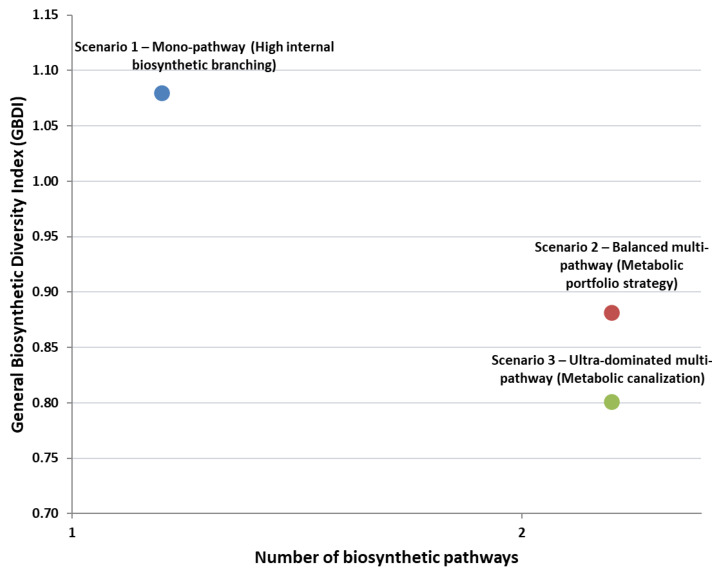
Conceptual behavior of the General Biosynthetic Diversity Index (GBDI) in limiting mixture scenarios.

**Figure 2:**
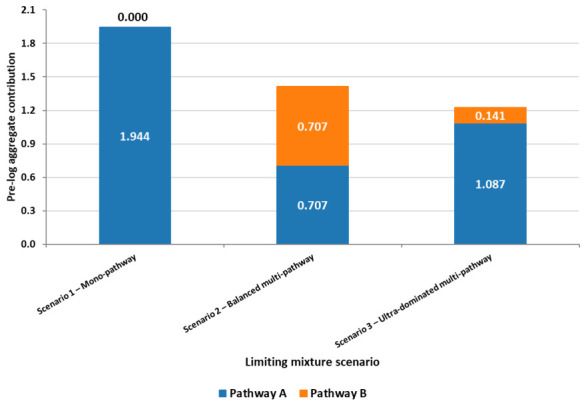
Decomposition of the pre-logarithmic GBDI aggregate into pathway-level contributions.

The ultra-dominated multi-pathway scenario produced the lowest GBDI value (0.800). In this example, route A represents the dominant hypothetical route and accounts for most of the mixture (*P*A = 0.90), whereas route B represents the minor route (*P*B = 0.10). Within route A, one metabolite dominates the entire profile (*p*_i_ = 0.85), limiting intrapathway branching despite the high route-level abundance. The minor route contributes little to the pre-logarithmic aggregate because its cumulative abundance is small. This pattern is consistent with metabolic canalization, in which biosynthetic investment is organized around the production of one principal compound rather than around broad molecular diversification. Such canalization may be adaptive when a single compound confers strong defensive or signaling efficacy, or when physiological constraints yield over structural diversification. The comparative behavior of these scenarios is shown in Figure 1, and the corresponding route-level decomposition is shown in Figure 2.

Together, the theoretical scenarios indicate that GBDI can separate three conditions that are often conflated by abundance-based indices: internal diversification within a dominant pathway, balanced allocation across pathways, and canalized production dominated by a single metabolite. This property supports the use of the index as a comparative descriptor of pathway-informed chemical organization, provided that it is interpreted together with route-level decomposition and dominance metrics.

### 2.3. Biosynthetic Architecture Across Plant Organs of Piper Rivinoides

**Figure 3:**
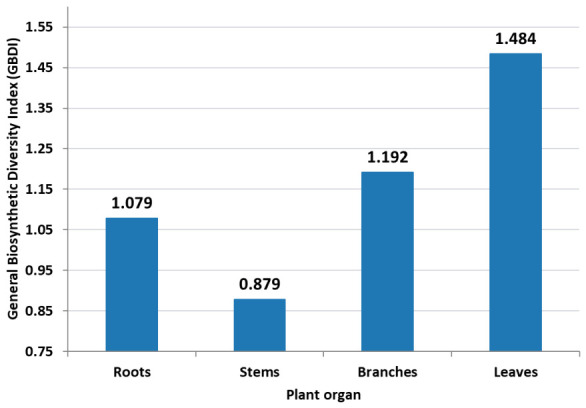
General Biosynthetic Diversity Index values calculated for essential oils from different organs of *Piper rivinoides*.

Application of GBDI to essential oils from different organs of *P. rivinoides* [19] revealed marked organ-specific differences in biosynthetic architecture. Leaves exhibited the highest GBDI value (1.484), followed by branches (1.192), roots (1.079), and stems (0.879) with α = 0.5 (for other α values see Table S2). Because GBDI integrates pathway-level allocation with intrapathway diversification, this ordering should not be interpreted as a simple ranking of chemical richness. Instead, it indicates that organs differ in the way their volatile chemical investment is distributed across biosynthetic routes and within those routes. The organ-specific GBDI values and their architectural interpretation are summarized in Table 3, and the visual pattern of value distribution is shown in Figure 3.

Leaves displayed the most diversified biosynthetic architecture. Their volatile profile combined a high monoterpene contribution with a substantial sesquiterpene fraction, and both terpene classes were internally diversified. This dual investment suggests that leaf metabolism is not restricted to a single canalized route but is organized as a chemically heterogeneous volatilome. Such architecture is consistent with the ecological role of leaves as the principal interface with herbivores, pathogens, solar radiation, and atmospheric stressors. A broadened volatile repertoire may increase functional redundancy, expand the range of deterrent or signaling activities, and improve responsiveness to fluctuating biotic interactions [32,33,34,35,36,37,38,39,40].

Branches showed an intermediate GBDI despite being strongly dominated by monoterpenes. This is an important result because it demonstrates that high pathway dominance does not necessarily imply low biosynthetic diversity. In branches, the elevated GBDI relative to stems indicates that the dominant monoterpene pathway is internally diversified. Thus, the architectural complexity of branches arises mainly from intrapathway branching rather than from balanced allocation among several biosynthetic routes.

Stems exhibited the lowest GBDI and the strongest canalization. Their volatile chemistry was dominated by arylpropanoid production, particularly by apiole-type compounds, with limited contribution from other routes. This architecture suggests concentrated metabolic investment in a restricted set of products. Ecologically, canalization in stems may reflect a strategy of constitutive protection in supporting tissues, where high yields of a few effective compounds may be more advantageous than broad chemical dispersion. From a phytochemical perspective, such canalized organs may be favorable for targeted isolation, standardization, and scale-up of dominant metabolites because chemical complexity is reduced.

Roots presented an intermediate-to-low GBDI and were also structured mainly by arylpropanoids, with secondary contributions from sesquiterpenes. This pattern suggests a more specialized belowground volatile architecture, potentially linked to antimicrobial activity, rhizospheric interactions, and protection against soil-borne organisms [41,42,43,44,45]. The root profile therefore illustrates a strategy in which chemical potency and route-level specialization may be prioritized over broad pathway diversification.

The organ-level results indicate that *P. rivinoides* modulates volatile biosynthetic architecture in a tissue-specific manner. Leaves represent a diversified aerial interface, branches represent a focused but internally branched monoterpene system, and stems and roots represent more canalized arylpropanoid-rich architectures. GBDI therefore captures an organizational dimension that is not accessible through a simple inspection of dominant compounds: it differentiates between pathway canalization and intrapathway diversification even when one route accounts for most of the mixture.

### 2.4. Ontogenetic Reorganization of Biosynthetic Architecture

The ontogenetic dataset showed a progressive increase in GBDI across developmental phases of *P. rivinoides*. GBDI increased from Phase I (0.936) to Phase V (1.443), indicating that the volatile biosynthetic architecture becomes increasingly branched during plant maturation. The fitted developmental trajectory was strong (R2 = 0.947; *p* = 0.0052), but, because only five ordered phases were available, it was treated as descriptive rather than definitive inferential evidence. Sensitivity analysis showed that the ontogenetic direction was stable under alpha = 0.25 and alpha = 0.50 and remained broadly consistent under alpha = 0.75, whereas alpha = 1.00 gave greater weight to dominance and produced a more conservative interpretation of sample ordering (Tables S3 and S4). The GBDI values and selected classical chemodiversity descriptors for each developmental phase are summarized in Table 4.

Phase I combined the lowest GBDI with low Shannon diversity and low evenness. This pattern is consistent with a highly canalized volatile mixture structured around a restricted set of major compounds. In biosynthetic terms, early-stage metabolism appears to favor concentrated investment over broad branching. Such a strategy may be advantageous during juvenile development, when structural tissues are still forming and metabolic resources may be constrained. Under this interpretation, early canalization reflects a metabolic economy strategy: a limited number of high-abundance compounds may provide essential protection with reduced biosynthetic dispersion [46,47,48,49].

Phase II showed a modest increase in both compositional and biosynthetic diversity, indicating the beginning of architectural expansion while retaining a strongly canalized profile. The transition from Phase II to Phase III was more pronounced. GBDI increased to 1.292, Shannon diversity reached approximately 2.54, Simpson diversity approached 0.89, and Pielou evenness reached 0.72. This phase therefore marks an important developmental transition in which the plant not only accumulates a more even mixture of metabolites but also redistributes metabolic investment across a broader biosynthetic network. The increasing participation of terpene pathways from this point onward supports the interpretation of a shift from phenylpropanoid-dominated early defense toward a terpene-enriched volatilome [19,33,46,47,48,49,50].

Phase IV was particularly informative because Shannon diversity decreased relative to Phase III (*H*^′^ approximately 1.91), whereas GBDI continued to increase to 1.387. This divergence demonstrates that compositional diversity and biosynthetic architecture are not equivalent. A mixture may become more dominated at the compound level while still retaining or increasing route-level organization and intrapathway branching. Therefore, the Phase IV pattern suggests selective reinforcement of certain volatile products without collapse of the underlying biosynthetic network.

**Figure 4:**
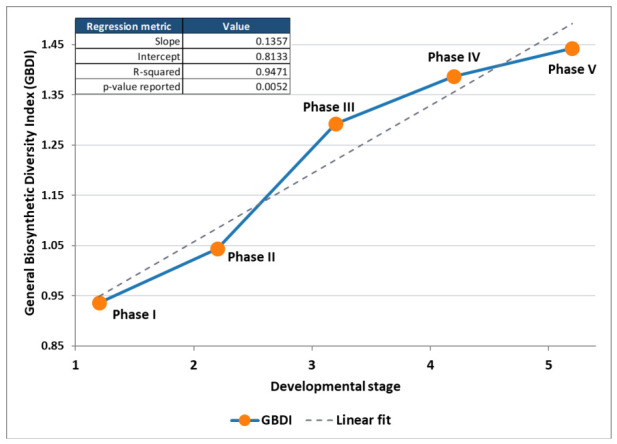
Ontogenetic trajectory of GBDI across five developmental phases of *Piper rivinoides*.

Phase V presented the highest GBDI and high classical chemical diversity values, with Shannon diversity increasing again to approximately 2.46 and Pielou evenness reaching 0.64. This mature stage thus combined compositional complexity with the most expanded biosynthetic architecture observed in the ontogenetic series. The original ontogenetic study reported 95 compounds across phases, eight compounds present in all phases, and 18 compounds shared between Phases IV and V, supporting the interpretation of late-stage expansion and partial stabilization of the volatile chemical space [19]. The overall ontogenetic trajectory and its linear tendency are shown in Figure 4.

To address the limited number of developmental phases, the revised analysis included rank-based tests such as Spearman’s rho and Kendall’s tau, robust slope estimation using the Theil-Sen estimator, permutation testing of the slope, and bootstrap confidence intervals (Note S2; Tables S5–S8). These procedures reduce dependence on normality assumptions and quantify uncertainty around the developmental increase in GBDI; nevertheless, the ontogenetic pattern should remain interpreted as descriptive support that requires expanded empirical validation (Tables S5–S8).

The ontogenetic trajectory has direct implications for chemical ecology and bioprospecting. Early phases, characterized by canalization, may facilitate the isolation of dominant compounds with high yield and lower chemical interference. Later phases, characterized by higher GBDI, may expand access to broader chemical space and increase the likelihood of detecting minor, rare, or structurally informative metabolites. Thus, developmental stages should be considered a strategic sampling variable rather than a nuisance factor in phytochemical studies.

### 2.5. Spatial Versus Ontogenetic Metabolic Plasticity

Comparison between organ-level and ontogenetic GBDI patterns reveals two different axes of volatile metabolic plasticity. Spatial variation among organs reflects modular specialization, whereas ontogenetic variation reflects directional developmental expansion. These axes are complementary rather than redundant.

Spatial variation does not follow a simple linear gradient. Leaves, branches, roots, and stems occupy distinct architectural positions that likely reflect tissue-specific ecological functions. Leaves exhibit a distributed architecture compatible with broad exposure to biotic and abiotic pressures, including herbivores, pathogens, radiation, and atmospheric stressors [19,32,33,37,38,39]. Branches exhibit intrapathway monoterpene diversification. Roots and stems exhibit stronger arylpropanoid canalization, compatible with targeted belowground and structural defense [19,37,41,43,44,45,51]. Thus, organ-level GBDI differences indicate functional compartmentalization of volatile biosynthesis.

Ontogenetic variation, by contrast, shows a directional increase in architectural complexity. Early phases are more canalized toward arylpropanoids, whereas later phases progressively incorporate broader terpene contribution and greater route-level branching [19,26,47,48,49]. This pattern suggests developmental reconfiguration of metabolic networks. The plant does not simply replace one dominant compound with another; instead, it modifies the hierarchical organization of volatile chemical investment.

From an evolutionary perspective, these two axes may represent complementary adaptive strategies. Organ-specific canalization can optimize chemical function in tissues where a targeted defense or structural role is advantageous, whereas developmental expansion can increase chemical flexibility as the plant becomes larger, more exposed, and more integrated into ecological interaction networks. GBDI is useful precisely because it resolves both modular specialization and directional developmental diversification within a single structural framework [8,19,26,50].

These patterns also define distinct bioprospecting windows. Arylpropanoid-rich canalized samples, especially stems, roots, and early developmental phases, may be prioritized when the objective is efficient extraction, targeted isolation, or standardization of dominant compounds with reduced chemical interference. Conversely, leaves and later ontogenetic phases represent exploratory targets because they combine higher GBDI with broader terpene participation and greater probability of detecting minor, rare, or structurally informative metabolites [27,28,29,30,31].

### 2.6. Relationship Between GBDI and Classical Chemodiversity Indices

**Figure 5:**
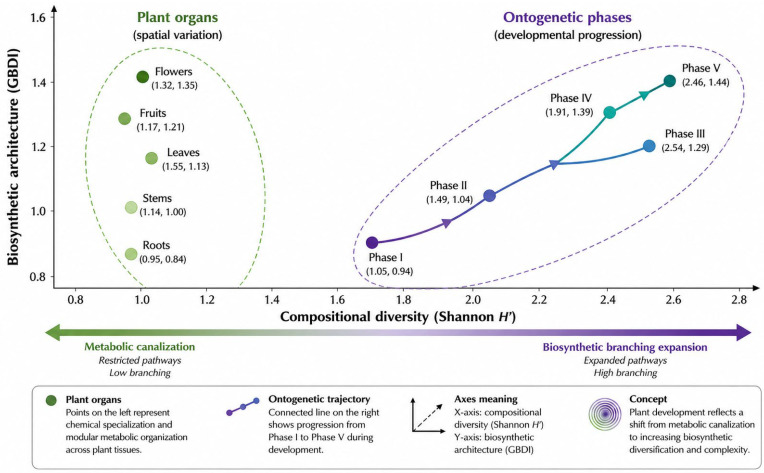
Conceptual integration of compositional chemical diversity and biosynthetic architecture using the revised Shannon diversity and GBDI values.

Classical chemical alpha-diversity descriptors remain essential for chemical ecology because they quantify the distribution of individual compounds within a mixture. Shannon diversity captures richness and proportional abundance, Simpson diversity is more sensitive to dominance, and Pielou evenness expresses how evenly abundance is distributed among the observed compounds [13,14,15]. However, these metrics do not incorporate biosynthetic attribution and therefore cannot distinguish whether compounds are distributed across several biosynthetic routes or concentrated within one internally diversified pathway [8,10,50]. This conceptual distinction is summarized in Figure 5, in which compositional diversity, represented by Shannon *H*^′^, is contrasted with biosynthetic architecture, represented by GBDI.

In the organ-level dataset, leaves exhibited the highest values of Shannon diversity (*H*^′^ = 3.48), Simpson diversity (1 − *D* = 0.93), and Pielou evenness (*J* = 0.73), indicating the most compositionally diverse and evenly distributed mixture among the examined organs. Roots also showed high compositional diversity (*H*^′^ = 3.00; *J* = 0.65), whereas stems showed lower Shannon diversity (*H*^′^ = 2.89) but relatively high evenness (*J* = 0.71), and branches presented the lowest Shannon diversity (*H*^′^ = 2.57) and Simpson diversity (1 − D = 0.87). Although these indices effectively describe compound-level distribution, they do not resolve whether the observed compounds are organized across many biosynthetic routes or concentrated within a few internally branched modules. Accordingly, the organ-related points shown in Figure 5 illustrate how tissue-level chemical specialization may occur within a more canalized region of the compositional–biosynthetic space.

The original organ-level study reported 111 compounds in essential oils from different organs, with leaves and roots showing the highest numbers of identified compounds (30 and 24, respectively) and 11 compounds shared between them [19]. These richness values are informative but insufficient to infer biosynthetic architecture. Roots, for example, may be compositionally rich while remaining metabolically canalized toward arylpropanoid production. For future GBDI applications, compound richness should be reported together with pathway richness, number of compounds per pathway, top-three or top-five cumulative abundance, *p*max, *P*max, Hpath, Hwithin,k, and C_k_(%). This set of outputs clarifies whether high GBDI derives from broad richness, pathway redistribution, or intrapathway branching.

The ontogenetic comparison further demonstrates the partial independence between compositional diversity and biosynthetic architecture. Phase I showed the lowest compositional diversity and biosynthetic organization (*H*^′^ approximately 1.05; 1 − D approximately 0.41; *J* approximately 0.36; GBDI = 0.936), indicating a highly dominated mixture structured around a restricted number of major metabolites and limited pathway diversification. Phase II showed modest expansion (*H*^′^ approximately 1.49; GBDI = 1.040), whereas Phase III showed a pronounced increase in both compositional and biosynthetic dimensions (*H*^′^ approximately 2.54; 1 − D approximately 0.89; GBDI = 1.292). Phase IV was especially informative because Shannon diversity decreased (*H*^′^ approximately 1.91) while GBDI continued to rise (1.387), demonstrating that compound-level dominance can increase without collapse of route-level organization. Phase V combined renewed compositional expansion (*H*^′^ approximately 2.46) with the highest GBDI (1.443), indicating mature-stage integration of compound-level complexity and biosynthetic architectural expansion [8,19,26,33,46,47,48,49,50].

These results support a conceptual separation between compositional chemodiversity and biosynthetic architecture. Shannon and Simpson indices primarily quantify how individual metabolites are distributed, whereas GBDI evaluates how metabolic investment is deployed across biosynthetic routes and how diversified those routes are internally. In ecological and evolutionary terms, this distinction corresponds to two different chemical strategies: compound-level heterogeneity and pathway-informed architectural complexity. Thus, Figure 5 provides a visual synthesis of the proposed interpretative framework, emphasizing that high compositional diversity does not necessarily imply high biosynthetic architectural complexity, and that GBDI adds a pathway-informed dimension to classical chemodiversity analysis.

### 2.7. Between-Pathway and Within-Pathway Diversity as Complementary Descriptors of GBDI

To make the interpretation of GBDI more explicit, two complementary Shannon-based descriptors were calculated from the same pathway-attributed abundance matrix. Between-pathway diversity, calculated using Equation (4), summarizes how evenly metabolic investment is distributed among biosynthetic routes and is expressed as an effective number of routes through the Hill-number transformation. Intrapathway diversity, calculated using Equation (5), normalizes compounds within each route and indicates whether a dominant biosynthetic pathway is chemically narrow or internally branched. Thus, these descriptors separate two components that are integrated by GBDI: allocation among routes and branching within routes. This distinction is critical because biosynthetic canalization and diversification can occur at different hierarchical levels of chemical organization [8,10,20,24,25,26,50].

For interpretation, Hpath should be read as a route-level allocation descriptor. Values approaching zero indicate that the effective number of biosynthetic routes approaches one, meaning that most metabolic investment is concentrated on a single route. Higher Hpath and ^1^Dpath values indicate more even distribution of abundance among routes, but they do not necessarily imply extensive molecular branching. In contrast, Hwithin,k and ^1^Dwithin,k evaluate the internal diversification of a given route after normalization within that route. Therefore, a low-Hpath/high-Hwithin,k profile indicates focused but branched metabolism, whereas a high-Hpath/low-Hwithin,k profile indicates broad allocation across routes but shallow diversification within each route. The interpretive combinations adopted in this study are summarized in Table 5 [13,14,15,16,52,53,54,55].

To separate canalization from diversification more explicitly, the interpretation was based on a set of companion indicators rather than on GBDI alone. Compound-level dominance identifies whether the mixture is controlled by one metabolite; pathway-level dominance identifies whether the mixture is controlled by one biosynthetic route; Hpath and ^1^Dpath quantify route-level dispersion; Hwithin,k and ^1^Dwithin,k quantify branching inside each route; and C_k_(%) identifies which biosynthetic class drives the pre-logarithmic GBDI aggregate. These indicators provide an operational diagnostic framework for distinguishing metabolic canalization, focused branching, and broadly distributed biosynthetic diversification (Table 6) [8,10,13,14,15,16,50,52,53,54,55,56,57].

This two-level framework prevents the misinterpretation of Hpath as a direct substitute for GBDI. Hpath measures how evenly abundance is allocated among biosynthetic routes, whereas GBDI integrates this allocation with the internal diversification of compounds produced by each route. Consequently, canalization should be inferred only when route-level concentration is accompanied by low intrapathway branching and high molecular dominance. Conversely, diversification may occur either through broad distribution among pathways or through extensive branching within a dominant pathway [8,10,20,24,25,26,50].

**Figure 6:**
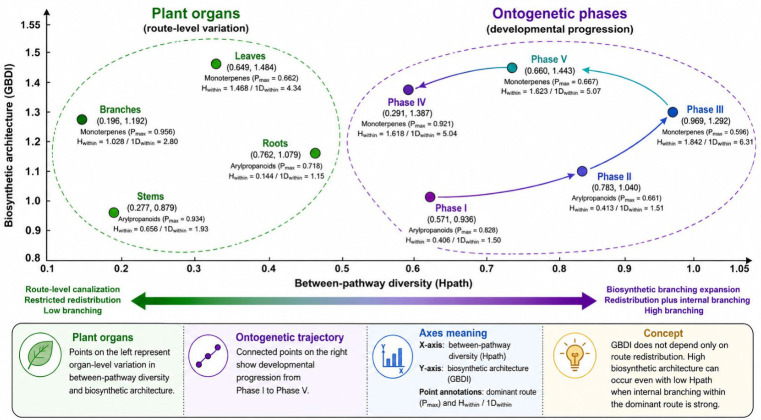
Integrated graphical synthesis of the biosynthetic architecture of *Piper rivinoides*, showing organ-level variation and ontogenetic progression based on GBDI and between-pathway diversity Hpath, with point labels indicating dominant biosynthetic routes and within-route descriptors.

As summarized in Table 7 and visualized in Figure 6, the integrated interpretation separates organ-level, ontogenetic, and dominant-route patterns by analytical scale. At the organ level, stems and roots represent arylpropanoid-centered canalization, branches represent a focused-but-branched monoterpene profile, and leaves represent the most diversified organ-level architecture. Across ontogeny, Phase I and Phase II retain arylpropanoid-centered canalization, Phase III marks the strongest route-level redistribution, and Phases IV and V indicate late monoterpene branching with sesquiterpene participation.

A clearer separation between canalization and diversification emerges when Hpath and Hwithin,k are interpreted jointly rather than sequentially. Hpath values close to zero indicate that metabolic investment is effectively concentrated in one biosynthetic route, while ^1^Dpath translates this entropy into the number of equally abundant pathways required to reproduce the observed allocation. However, low Hpath should not be interpreted automatically as low chemical diversification. If Hwithin,k is high, the sample expresses a dominant biosynthetic route through several relevant metabolites, indicating intraroute branching rather than architectural impoverishment. This distinction is consistent with the concept that plant secondary metabolism may be modular, with ecological diversification occurring either by reallocation among pathways or by elaboration within one pathway family [20,24,25,26,50,51].

As integrated in Table 7 and synthesized in Figure 6, stems and roots exemplify canalization more clearly than branches because their arylpropanoid-rich profiles combine high *P*max with limited or moderate intrapathway diversification. Roots, in particular, showed high Hpath but very low Hwithin,k within arylpropanoids, showing that apparent route dispersion can coexist with a narrow dominant chemical module. Branches and Phase IV represent a different architecture: both had low Hpath and high Pmax, but high Hwithin,k within monoterpenes. These samples should therefore be interpreted as focused-but-branched systems rather than simply canalized systems. Phase III showed the strongest between-pathway redistribution, whereas Phase V combined high GBDI with sustained monoterpene branching and increased sesquiterpene participation. Thus, the expanded descriptor set separates three states that would otherwise be conflated: route canalization, intrapathway diversification, and broad biosynthetic diversification [8,10,13,14,15,16,50,52,53,54,55,56,57]. These relationships are synthesized in Figure 6, which integrates organ-level variation and ontogenetic progression in the same GBDI-Hpath space.

This interpretation strengthens the ecological and bioprospecting relevance of the GBDI framework. Canalized profiles, especially those with high *p*max, high *P*max, low Hpath, and low Hwithin,k, may be advantageous when a small set of metabolites provides efficient constitutive defense or when the objective is targeted isolation of high-yield compounds. Diversified profiles, especially those with high GBDI and elevated Hwithin,k or Hpath, may indicate broader chemical space exploration, higher probability of detecting structurally varied constituents, and greater potential for multifunctional ecological interactions. Therefore, Hpath should be used as a diagnostic measure of route-level allocation, while Hwithin,k should be used as the key indicator of internal biosynthetic branching [27,28,29,30,31,39,40,50].

Numeric values displayed in Figure 6 and used to generate the condensed interpretations in Table 7 were calculated after normalizing route totals or within-route compound abundances to unity. Monoterpenes combine non-oxygenated and oxygenated monoterpenes, sesquiterpenes combine non-oxygenated and oxygenated sesquiterpenes, and arylpropanoids were treated as a separate route. These calculations are implemented in the automated spreadsheet (see Supplementary Material).

As summarized in Table 8, a supplementary correlation matrix including GBDI, Shannon diversity, Simpson diversity, Pielou evenness, richness, Hpath, pmax, and Pmax was prepared to evaluate the empirical relationship between GBDI and classical descriptors. The objective was not to force convergence among metrics, but to test whether GBDI provides information beyond simple compositional diversity in this dataset. The automated spreadsheet provides a reproducible structure for these calculations (see Supplementary Material—Notes S1 and S2; Tables S1–S9).

### 2.8. Conceptual Positioning Relative to RaoQ, Functional Hill Numbers, and Bray-Curtis Dissimilarity

Several established ecological metrics may appear conceptually close to GBDI, particularly Rao’s quadratic entropy (RaoQ), functional Hill numbers, and Bray-Curtis dissimilarity. A clear conceptual delimitation is therefore necessary to avoid interpreting GBDI as a reformulation of existing diversity measures. RaoQ combines relative abundance with pairwise dissimilarities among components, effectively quantifying the abundance-weighted expectation of dissimilarity between two randomly drawn elements [56]. In chemical applications, those dissimilarities may be based on molecular fingerprints, physicochemical descriptors, structural classes, or functional traits. Its value therefore depends on the selected distance matrix, whereas GBDI does not require pairwise molecular distances. GBDI uses abundance and biosynthetic route attribution to quantify how chemical investment is hierarchically organized.

Functional Hill numbers estimate the effective number of functionally distinct entities within a chosen trait space [52,53,54,55]. These metrics are powerful and flexible, but their interpretation remains tied to effective variety under a similarity or dissimilarity structure. GBDI answers a different question. It does not estimate the effective number of compounds or pathways, does not require the choice of a diversity order parameter q, and does not depend on a trait or similarity matrix. Instead, it quantifies the degree to which observed abundance is deployed across biosynthetic routes and diversified internally. Therefore, two mixtures may have similar functional Hill diversity, but different GBDI values if their metabolites occupy similar structural space while being produced through different biosynthetic deployment patterns.

Bray-Curtis dissimilarity operates at another level because it is a between-sample measure of compositional turnover [57]. It addresses how different two mixtures are from each other, whereas GBDI describes the internal biosynthetic architecture of one mixture. Two samples may be highly dissimilar by Bray-Curtis yet present similar GBDI values if both are similarly organized across routes. Conversely, samples may be compositionally similar by Bray-Curtis but differ in GBDI if pathway-level allocation shifts without large changes in the total abundance vector. The conceptual distinctions among these metrics are summarized in Table 9.

These metrics should therefore be treated as complementary rather than competing. RaoQ and functional Hill diversity quantify structural or functional dissimilarity within a chosen trait space; Bray-Curtis quantifies compositional turnover between samples; and GBDI quantifies pathway-informed organization within samples [52,53,54,55,56,57]. Their joint use can disentangle molecular heterogeneity, compositional beta-diversity, pathway-level investment, and biosynthetic branching. Integrating GBDI with Bray-Curtis, for example, would allow simultaneous assessment of compound-level turnover and biosynthetic turnover by computing dissimilarities on pathway-aggregated abundance vectors or route-level GBDI contributions.

For this reason, GBDI should be interpreted as a complementary pathway-informed descriptor of metabolic architecture rather than as a substitute for RaoQ, functional Hill diversity, or Bray-Curtis dissimilarity. Claims of independence from existing metrics should be evaluated empirically for each dataset through correlation analysis, multivariate ordination, variance partitioning, or simulation. In natural product discovery, coupling GBDI with molecular networking, dereplication, and cheminformatic descriptors may generate a multiscale framework linking biosynthetic organization to chemical-space prioritization [27,28,29,30,31].

### 2.9. Analytical Safeguards, Sensitivity Analysis, and Null Expectations

Robust interpretation of GBDI depends on three analytical decisions: compound detection and filtering, biosynthetic route assignment, and the value of α. Peak areas must be normalized to sum to one within each sample, and the filtering threshold must be reported because inclusion of trace compounds can influence concave diversity metrics. Route assignment is particularly important because metabolites may arise from shared precursors, mixed origins, or uncertain annotations [10,11,12,20,23,24,25,26]. Therefore, the pathway-attributed matrix should document the rationale and confidence level for each assignment; when alternative assignments are plausible, GBDI should be recalculated under those alternatives to test ranking stability and interpretation.

The recommended α-grid for sensitivity analysis is summarized in Table 10 and was applied in this revision. Values below 0.50 increased the influence of minor branches, α = 0.50 provided balanced concave weighting, α = 0.75 retained partial dominance control, and α = 1.00 represented a linear endpoint dominated by major biosynthetic investments. The organ-level rankings were more sensitive to alpha than the ontogenetic trajectory, indicating that conclusions should be reported together with the chosen parameter and its sensitivity output. The automated spreadsheet allows users to compare alpha values and inspect the stability of sample rankings (see Supplementary Material, Tables S1–S4).

Null models can add an inferential layer to GBDI. Pathway-label permutation asks whether the observed association between metabolites and biosynthetic routes matters. Abundance permutation asks whether the empirical abundance structure matters. Within-route permutation, preserving *P*_k_ while shuffling abundances inside routes, asks whether intrapathway diversification is higher or lower than expected given route-level investment. Standardized effect sizes against these null distributions would allow one to distinguish architecturally overdispersed mixtures from canalized mixtures. This approach is consistent with broader ecological diversity frameworks that compare observed diversity patterns with null expectations [52,53,54,55,56,57]. The standardized effect-size expression for comparing observed GBDI values with null expectations is presented in Equation (6).

(6) $$SES=\frac{GBDI_{obs}-average(GBDI_{null})}{DP(GBDI_{null})}$$

Positive standardized effect sizes would indicate greater biosynthetic architectural diversification than expected under a null model, whereas negative values would indicate stronger canalization. For example, leaves would be interpreted as architecturally overdispersed only if their observed GBDI exceeds null expectations, whereas stems would be interpreted as canalized if their GBDI falls below the null distribution. This approach would strengthen the statistical interpretation of GBDI beyond descriptive ranking.

### 2.10. Implications for Evolutionary Ecology, Bioprospecting, and Natural Product Discovery

The implications of GBDI should be framed within the limits of a proof-of-concept metric. The index does not demonstrate ecological or evolutionary mechanisms by itself; instead, it generates quantitative hypotheses about whether a chemical phenotype is concentrated in a few biosynthetic routes, internally branched within one route, or distributed among multiple routes. This narrower framing avoids treating the metric as direct evidence of adaptation or innovation and aligns the interpretation with the empirical scope of the present dataset [8,58,59].

In this manuscript, “biosynthetic architecture” refers to the abundance-weighted organization of metabolites within and among assigned biosynthetic routes; “expanded biosynthetic space” refers to higher route participation and/or greater intrapathway branching; and “chemical-space exploration” is used only as a bioprospecting-oriented hypothesis about the probability of encountering broader structural variation. These terms are descriptive and operational, not direct measurements of evolutionary novelty or pathway flux.

**Figure 7:**
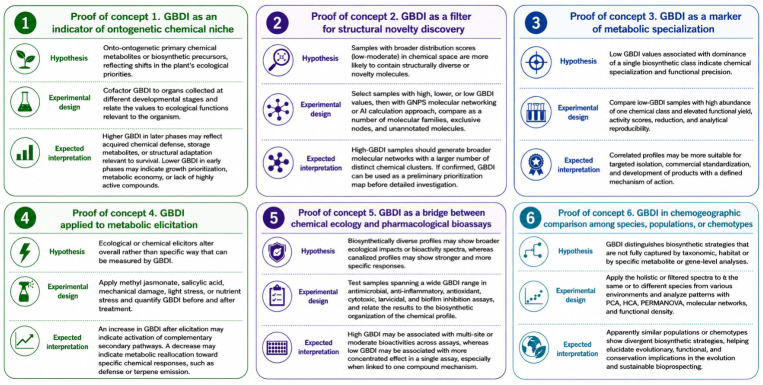
Proof-of-concept framework for applying the GBDI to ecological, evolutionary and bioprospecting studies of plant chemodiversity.

This interpretation allows chemodiversity to be treated as a multidimensional descriptor that can be decomposed into pathway breadth, route-level investment, intrapathway branching, compound dominance, and functional dispersion among metabolic classes. Accordingly, GBDI contributes to transforming verbal hypotheses about chemical diversity into quantitative hypotheses that can be tested among organs, ontogenetic stages, chemotypes, ecotypes, populations, or phylogenetic lineages when adequate sampling is available [7,8,50,60]. These applications are synthesized in Figure 7.

Profiles with high GBDI should therefore be interpreted as candidates for broader or more branched biosynthetic organization, whereas profiles with low GBDI may indicate canalization, specialization, or concentration of dominant markers. Both patterns can be biologically and technologically relevant, depending on the research objective [4,12,24,25,26,51].

In bioprospecting, GBDI may serve as a complementary prioritization criterion. High values can guide exploratory screening for chemically heterogeneous samples, intermediate values can support the search for biosynthetically related compound series, and low values can support targeted isolation, standardization, quality control, and scalable acquisition of dominant markers [27,28,29].

The proposed application is relevant because natural products are both potential pharmacological candidates and ecological traits. Incorporating biosynthetic organization into bioprospecting recognizes that sample selection can consider not only bioactivity or abundance, but also the metabolic structure that conditions compound production [2,3,61].

Biosynthetically broad profiles are not intrinsically superior to canalized profiles. The former may be useful for exploratory discovery, whereas the latter may be preferable for reproducible extraction, standardization, and technological development. This distinction links discovery-oriented and development-oriented stages of natural product research [24,27,28,29,51].

GBDI can also be applied to induced-response experiments, in which elicitors, herbivory, light, water deficit, competition, or microbial interactions alter pathway allocation. In this context, the index can help determine whether a treatment makes the chemical phenotype more canalized, more internally branched, or more distributed across biosynthetic routes [8,37,42,62,63].

## 3. Materials and Methods

### 3.1. Study Design

This study was conducted as a methodological proof of concept for the General Biosynthetic Diversity Index (GBDI), a pathway-informed metric designed to describe the biosynthetic organization of plant chemical mixtures. The workflow included: chemical abundance matrix organization, metabolite assignment to biosynthetic routes, GBDI calculation, route-level decomposition, comparison with classical chemodiversity and chemical-diversity metrics, sensitivity analysis, null-model testing, and empirical application to *Piper rivinoides* Kunth (Piperaceae).

### 3.2. Dataset

The empirical dataset was obtained from a previously published study on the volatile composition of *P. rivinoides* essential oils [19]. The dataset included mean relative-abundance profiles from roots, stems, branches, and leaves, as well as five ontogenetic phases. Phase I corresponded to juvenile plants approximately 25 cm tall with unbranched herbaceous stems. Phase II included plants approximately 40 cm tall with initial branching. Phase III included plants approximately 70 cm tall with developed herbaceous branching. Phase IV corresponded to plants approximately 2 m tall with lignified stems and multiple branches. Phase V corresponded to fully developed plants approximately 7 m tall with thick lignified stems and extensive branching [19]. No new botanical sampling, extraction, chromatographic analysis, or taxonomic identification was performed. The original dataset was used as published, including GC-FID relative abundances, compound annotations, and mean abundance profiles [19].

### 3.3. Data Preparation and Normalization

Chemical profiles were organized as abundance matrices in which rows represented samples and columns represented metabolites. Relative abundances were expressed as proportions. When original values were reported as percentages, they were divided by 100.

For each sample, the abundance vector was defined as: $p=(p_{1},p_{2},\ldots ,p_{n})$ where $p_{i}$ is the relative abundance of metabolite i, and n is the number of retained metabolites. Abundances were normalized so that: $\sum_{i=1}^{n}p_{i}=1$. Compounds without reliable structural annotation, without interpretable biosynthetic assignment, or below the inclusion threshold of the original dataset were excluded from the main matrix.

### 3.4. Biosynthetic Assignment

Each retained metabolite was assigned to a biosynthetic route according to chemical class, precursor origin, and accepted biosynthetic interpretation.

Biosynthetic assignment can be performed at the level of major precursor routes (e.g., acetate–mevalonate or methylerythritol 4-phosphate (MEP) for terpenoids, acetate for fatty-acid-derived, shikimate for phenylpropanoids/lignoids/coumarins/gallic tannins, and other aromatic compounds **C6**-**C1**/**C6**-**C2**/**C6**-**C3**/**C6**-**C4**, mixed-origin acetate-shikimate for flavonoids/condensed tannins), as well as for different nitrogen-containing compounds (e.g., alkaloids in which precursors are lysine or ornithine, tryptophane, tyrosine or phenylalanine) [9,12]. We suggest using a more granular level if justified, mainly for comparison of chemotypes, geotypes, chronotypes, and ecotypes (e.g., non-oxygenated/oxygenated monoterpenes; non-oxygenated/oxygenated sesquiterpenes; phytane, clerodane, labdane, taxane, pimaran, and kaurane diterpenes; ursane, oleanane, lupane, friedelane, taraxastane, and dammarane triterpenes) [9,12].

For the *P. rivinoides* dataset, compounds were grouped into the main volatile biosynthetic classes represented in the dataset, including monoterpenes, sesquiterpenes, arylpropanoids, and other minor volatile classes when applicable. Each compound was assigned to one primary route, denoted as k(i). For metabolites with mixed or ambiguous biosynthetic origins, the assignment followed the most widely accepted precursor-route interpretation for the compound class, and alternative assignments can be tested in sensitivity analyses. The total abundance of each biosynthetic route was calculated as: $P_{k}=\sum_{i\in k}p_{i}$ where $P_{k}$ is the cumulative abundance of route k. The same classification criteria were applied to all samples, organs, and ontogenetic phases [20,21,22,23].

### 3.5. GBDI Calculation

GBDI was calculated according to Equation (1). The index combines compound-level abundance with the total abundance of the biosynthetic route to which each compound belongs. The scaling parameter α was fixed at 0.5 in the main analysis and then evaluated through a sensitivity grid (0.25, 0.50, 0.75, and 1.00) (Note S1; Table S1).

The calculation followed this sequence: normalization of relative abundances, assignment of metabolites to biosynthetic routes, calculation of $P_{k}$, computation of compound–route contributions, summation of contributions, and logarithmic transformation.

GBDI was interpreted as a comparative descriptor of abundance-weighted biosynthetic architecture. It was not interpreted as a direct measure of metabolic flux, enzymatic activity, gene expression, or pathway rate.

### 3.6. Route-Level Decomposition

The pre-logarithmic GBDI aggregate was decomposed into route-level contributions using Equation (2). The proportional contribution of each route was calculated using Equation (3).

The resulting route-level contribution matrix was organized by sample and biosynthetic route. This matrix was used to identify which pathways contributed most strongly to each GBDI value and to generate stacked contribution plots.

### 3.7. Between-Pathway and Intrapathway Diversity

Two complementary pathway-level descriptors were calculated. Between-pathway diversity was calculated using Equation (4) and expressed the effective number of biosynthetic routes represented in each sample. Intrapathway diversity was calculated using Equation (5) after conditional normalization of compounds within each route.

These descriptors were used to distinguish diversity arising from allocation among pathways from diversity arising from compound diversification within pathways.

### 3.8. Classical Chemodiversity Metrics

Classical chemodiversity metrics were calculated from the compound-level abundance matrix.

Shannon diversity was calculated as [13,19]:

(7) $$H^{\prime }=-\sum_{i=1}^{n}p_{i}ln(p_{i})$$

Simpson diversity was calculated as [14,19]:

(8) $$1-D=1-\sum_{i=1}^{n}p_{i}^{2}$$

Pielou evenness was calculated as [15,19]:

(9) $$J=\frac{H^{\prime }}{\ln(S)}$$

where S is the number of metabolites retained.

Shannon and Simpson indices were also transformed into effective numbers [14,15,19]:

(10) D1=exp(H′); D2=1∑i=1npi2

These metrics were used to compare GBDI with conventional descriptors of richness, dominance, and evenness.

### 3.9. Dominance and Canalization Descriptors

Compound-level and pathway-level dominance [20,21,22,23] were calculated as:

(11) $$p_{max}=max(p_{i});P_{max}=max(P_{k})$$

Cumulative dominance was calculated using the three and five most abundant compounds:

(12) $$Top3=\sum_{r=1}^{3}p_{\left(r\right)};Top5=\sum_{r=1}^{5}p_{\left(r\right)}$$

where $p_{\left(r\right)}$ is the abundance of the compound ranked in position *r*.

Berger–Parker dominance [32,33] was defined as: $d_{BP}=p_{max}$.
  The chemical Gini coefficient was calculated as:

(13) $$G=\frac{\sum_{i=1}^{n}\sum_{j=1}^{n}|p_{i}-p_{j}|}{2n\sum_{i=1}^{n}p_{i}}$$

The GBDI-to-dominant-compound ratio was calculated as:

(14) $$R_{GBDI/pmax}=\frac{GBDI}{p_{max}}$$

These descriptors were used to evaluate compound dominance, pathway dominance, and chemical canalization.

### 3.10. Hypothetical Scenarios

GBDI was first evaluated using three hypothetical chemical architectures: a mono-pathway mixture with internal diversification, a balanced multi-pathway mixture, and an ultra-dominated multi-pathway mixture. All scenarios were analyzed using the same calculation protocol and α=0.5. Route-level contributions were calculated using Equations (2) and (3).

### 3.11. Rao’s Quadratic Entropy and Bray–Curtis Dissimilarity

Rao’s quadratic entropy can be calculated as [56]:

(15) $$Q=\sum_{i=1}^{n}\sum_{j=1}^{n}p_{i}p_{j}d_{ij}$$

where $d_{ij}$ represents the chemical, structural, biosynthetic, or functional dissimilarity between metabolites i and j.

Bray–Curtis dissimilarity between samples a and b can be calculated as [57]:

(16) $$BC_{ab}=\frac{\sum_{i=1}^{n}|x_{ia}-x_{ib}|}{\sum_{i=1}^{n}(x_{ia}+x_{ib})}$$

These metrics were used as complementary descriptors of dissimilarity-weighted diversity and compositional turnover.

### 3.12. Sensitivity Analysis and Null-Model Analysis

Sensitivity analysis was performed by recalculating GBDI using α values of 0.25, 0.50, 0.75, and 1.00 for all organ-level and ontogenetic comparisons (See Supplementary Material, Tables S2 and S3). Rank stability among organs and ontogenetic phases was compared across alpha values. Permutation-based null models were used to compare observed GBDI values with randomized expectations [52,53,54,55,56,57]. Three models were applied: randomization of pathway labels while preserving abundance values, randomization of abundance values while preserving pathway labels, and randomization of abundances within each pathway while preserving total pathway abundance.

Standardized effect sizes were calculated according to Equation (6). Two-tailed permutation *p*-values were calculated as:

(17) $$p_{perm}=\frac{1+\sum_{b=1}^{B}I(|{\mathrm{GBDI}}_{b}-\mu_{null}|\geq |{\mathrm{GBDI}}_{obs}-\mu_{null}|)}{B+1}$$

where B is the number of permutations, ${\mathrm{GBDI}}_{b}$ is the permuted value, ${\mathrm{GBDI}}_{obs}$ is the observed value, $\mu_{null}$ is the mean of the null distribution, and I is the indicator function. For each null model, 9999 permutations were performed.

### 3.13. Ontogenetic and Correlation Analysis

Ontogenetic phases were treated as ordered developmental stages from Phase I to Phase V. The relationship between developmental stage and GBDI was evaluated using ordinary least squares regression, Spearman correlation, Kendall tau correlation, Theil–Sen slope estimation, permutation testing, and bootstrap confidence intervals. Because only five ontogenetic phases were available, these analyses were interpreted descriptively.

A correlation matrix was calculated to evaluate the association between GBDI and classical descriptors, including Shannon diversity, Simpson diversity, Pielou evenness, compound richness, between-pathway diversity, compound-level dominance, and pathway-level dominance. Pearson correlation was used for linear associations. Correlations were interpreted descriptively due to the limited number of empirical samples.

### 3.14. Computational Implementation, Reproducibility, and Reporting Standards

All calculations were performed from pathway-attributed abundance matrices. For each sample, the same workflow was applied: abundance normalization, pathway summation, GBDI calculation, route-level decomposition, chemodiversity metrics, dominance descriptors, sensitivity analysis, and null-model testing.

An automated spreadsheet was prepared as Supplementary Material. The spreadsheet allows users to input metabolite abundances and biosynthetic pathway labels and obtain GBDI values, route-level contributions, pathway diversity descriptors, dominance metrics, sensitivity outputs, and null-model summaries.

Applications of GBDI should report the analytical platform, normalization procedure, inclusion threshold, compound-identification criteria, biosynthetic-assignment criteria, alpha value, and full pathway-attributed abundance matrix.

When biological or analytical replicates are available, standard deviations, confidence intervals, detection limits, quantification limits, and annotation confidence levels should also be reported. When only mean abundance profiles are available, GBDI and derived descriptors should be interpreted as descriptive comparative measures.

### 3.15. AI Use

During the preparation of this manuscript, the authors used ChatGPT (Version 5.4.), developed by OpenAI, for language refinement, spelling corrections, and support in the preparation of Figure 5, Figure 6 and Figure 7. The authors reviewed and edited all generated outputs and took full responsibility for the content of this publication. The authors also wish to acknowledge the traditional and enchanted guardians of planet Earth, whose strength inspired the systematization of these data and whose presence reaffirms the harmony of nature as the foundation of the existence of all beings.

## 4. Conclusions

GBDI is a pathway-informed descriptor of abundance-weighted biosynthetic architecture. The proof-of-concept analyses show that the index combines two components that are not jointly represented by classical abundance-based metrics: allocation of chemical abundance among biosynthetic routes and diversification of compounds within those routes. The limiting scenarios support its interpretive use for distinguishing canalized mixtures, internally branched single-route mixtures, and more broadly distributed multi-route mixtures.

In the empirical *P. rivinoides* dataset, GBDI described organ- and development-related differences in volatile biosynthetic organization. Leaves presented the highest organ-level architectural diversity, branches showed focused monoterpene branching, and stems and roots showed more canalized arylpropanoid-rich profiles. The ontogenetic increase in GBDI suggests developmental expansion of biosynthetic branching, but this interpretation remains descriptive because it is based on five ordered phases and should be validated using expanded datasets.

The revised framework therefore positions GBDI as a complementary metric, not a replacement for Shannon, Simpson, Pielou, RaoQ, functional Hill numbers, or Bray-Curtis dissimilarity. Future applications should report route-level decomposition, between- and within-pathway diversity, dominance metrics, α-sensitivity, pathway-assignment criteria, and, when possible, null-model or independent-dataset validation. This reporting standard will clarify whether high GBDI values reflect broad cross-pathway allocation, strong intrapathway branching, or both.

## Figures and Tables

**Table 1:** Conceptual matrix for interpreting the General Biosynthetic Diversity Index in relation to molecular dominance, pathway dominance, and biosynthetic architecture.

GBDI Pattern	Compound-Level Dominance (*p*max)	Pathway-Level Dominance (*P*max)	Biosynthetic Interpretation	Architectural Meaning
Low GBDI	High $p_{max}$	High *P*max	Strong canalization toward one major compound within a dominant biosynthetic route.	Chemically narrow architecture, with concentrated metabolic investment and low branching.
Low GBDI	High $p_{max}$	Low or moderate *P*max	One compound dominates the mixture, although total biosynthetic allocation may be distributed across more than one route.	Molecular specialization is stronger than pathway-level specialization.
Low-to-moderate GBDI	Low or moderate $p_{max}$	High *P*max	Several compounds may occur, but most derive from the same predominant route.	Canalization at the pathway level with some intrapathway diversification.
Moderate GBDI	Moderate $p_{max}$	Moderate *P*max	No single compound or biosynthetic route completely dominates, but diversification remains limited.	Intermediate architecture, suggesting partial branching or transitional metabolic organization.
High GBDI	Low $p_{max}$	High *P*max	Many relevant compounds occur within a dominant biosynthetic route.	Focused but branched metabolism, characterized by high intrapathway diversification.
High GBDI	Low $p_{max}$	Low or moderate *P*max	Compounds are distributed across different biosynthetic routes without strong molecular predominance.	Broadly diversified biosynthetic architecture, with distributed metabolic investment.
High GBDI	Moderate $p_{max}$	Low *P*max	Some compounds remain abundant, but pathway allocation is not concentrated on a single route.	Diversified architecture with partial dominance, indicating coexistence between specialization and branching.
Very high GBDI	Low $p_{max}$	Low *P*max	Several compounds contribute substantially and are distributed across multiple biosynthetic routes.	Highly diversified multi-route biosynthetic architecture, characterized by low canalization and high metabolic branching.

**Table 2:** Limiting mixture scenarios used to examine GBDI behavior.

Scenario	Relative Architecture	Pre-Log Aggregate	GBDI (α = 0.5)	Interpretation
1. Mono-pathway branched	One pathway (A); four metabolites with abundances 0.40, 0.30, 0.20, and 0.10	1.944	1.079	Focused but internally diversified biosynthetic module
2. Balanced multi-pathway	Two pathways (A and B) with equal allocation; four metabolites with abundances 0.25 each	1.414	0.881	Portfolio-like allocation across routes, but lower route-level investment per pathway
3. Ultra-dominated multi-pathway	Two pathways (A and B); one dominant route and one dominant metabolite (A = 0.85, 0.05; B = 0.06, 0.04)	1.228	0.800	Metabolic canalization toward one principal product

**Table 3:** Organ-specific GBDI values and architectural interpretation in *Piper rivinoides* essential oils [19].

Organ	GBDI	Dominant Architectural Feature	Interpretation
Roots	1.079	Predominant arylpropanoid allocation with secondary sesquiterpene contribution	Belowground chemistry characterized by partial canalization and moderate branching
Stems	0.879	Strong arylpropanoid canalization, especially toward apiole-type chemistry	Narrow architecture consistent with targeted structural or constitutive defense
Branches	1.192	Very high monoterpene pathway allocation with internal route diversification	Focused but branched terpene architecture
Leaves	1.484	Substantial monoterpene and sesquiterpene contributions with internal diversification	Most distributed and architecturally diversified organ-level volatilome

**Table 4:** Ontogenetic variation in GBDI and classical chemodiversity descriptors.

Phase	GBDI	Shannon *H*^′^	Pielou *J*	Architectural Interpretation
I	0.936	1.05	0.36	Strong early canalization, mainly associated with arylpropanoid-dominated chemistry
II	1.040	1.49	0.45	Initial expansion of chemical and biosynthetic complexity, but still pathway-dominated
III	1.292	2.54	0.72	Marked redistribution of metabolic investment and increased route-level diversification
IV	1.387	1.91	0.49	Higher GBDI despite lower Shannon diversity, indicating biosynthetic organization beyond compositional evenness
V	1.443	2.46	0.64	Maximum observed architectural expansion and late-stage stabilization of volatile chemical space

**Table 5:** Conceptual matrix for interpreting between-pathway and intrapathway diversity in relation to biosynthetic canalization and diversification.

Between-Pathway Diversity	Within-Pathway Diversity	Interpretation
Low Hpath	Low intrapathway diversity	Extreme canalization: dominance of one route and one or few compounds.
Low Hpath	High intrapathway diversity	Diversification within a dominant route; focused but branched metabolism.
High Hpath	Low intrapathway diversity	Allocation across routes, but shallow diversification within each route.
High Hpath	High intrapathway diversity	Broadly diversified biosynthetic architecture with multiple branched routes.

**Table 6:** Operational indicators to distinguish biosynthetic canalization from diversification.

Analytical Dimension	Indicator	Canalization Pattern	Diversification Pattern
Compound-level concentration	*p*max; Top 3 or Top 5 abundance	High *p*max or high cumulative abundance of few compounds.	Lower dominance and larger contribution of several metabolites.
Pathway-level concentration	*P*max	High *P*max, indicating concentration of abundance in one route.	Lower *P*max, indicating more distributed route-level allocation.
Between-pathway allocation	Hpath; ^1^Dpath	Low Hpath and effective number close to one route.	High Hpath and higher effective number of routes.
Intrapathway branching	Hwithin,k; ^1^Dwithin,k	Low Hwithin,k, indicating one or few products inside a route.	High Hwithin,k, indicating several relevant products inside a route.
Route-level contribution to GBDI	C_k_(%)	One route accounts for most of the pre-logarithmic GBDI aggregate.	Several routes contribute materially to the aggregate, or one route is internally highly branched.
Architecture under dominance	GBDI relative to *p*max and *P*max	Low GBDI with high *p*max and high *P*max indicates canalization.	High GBDI despite high *P*max indicates focused intrapathway branching.

**Table 7:** Integrated interpretation of organ-level and ontogenetic biosynthetic architecture in *Piper rivinoides*.

Analytical Scale	Sample/Phase	Integrated Interpretation
Organ-level variation	Stems	Most canalized organ-level profile, characterized by restricted route allocation and a narrow apiole-dillapiole arylpropanoid module.
Organ-level variation	Roots	Comparatively broad route allocation but modest GBDI because the dominant arylpropanoid fraction remains close to apiole-driven canalization.
Organ-level variation	Branches	Focused-but-branched monoterpene architecture, with elevated GBDI despite low route-level dispersion.
Organ-level variation	Leaves	Most diversified organ-level profile, combining distributed terpene allocation and strong internal branching.
Ontogenetic progression	Phase I	Early arylpropanoid canalization with limited terpene contribution and low intrapathway branching.
Ontogenetic progression	Phase II	Transitional profile with increased terpene contribution, but still centered on a chemically narrow arylpropanoid core.
Ontogenetic progression	Phase III	Strongest route-level redistribution, accompanied by internal monoterpene diversification.
Ontogenetic progression	Phase IV	Strong monoterpene dominance with high intrapathway branching, indicating focused branching rather than simple canalization.
Ontogenetic progression	Phase V	Mature profile with high GBDI, sustained monoterpene branching, and expanded sesquiterpene participation.

**Table 8:** Companion descriptors used to evaluate the relationship between GBDI and classical chemodiversity metrics.

Metric	Primary Dimension Quantified	Relationship with GBDI
Shannon *H*^′^	Compound-level compositional diversity	May correlate with GBDI when compound diversity reflects pathway branching, but does not encode pathway attribution
Simpson 1 − D	Dominance/equitability at compound level	Useful companion for detecting whether GBDI is high despite compound dominance
Pielou *J*	Evenness relative to observed richness	Can diverge from GBDI when compounds are evenly distributed within one dominant route
Richness	Number of detected or retained compounds	Does not distinguish compounds from one route versus several routes
Hpath	Diversity among biosynthetic routes	Complements GBDI by isolating between-pathway allocation
*p*max	Dominance of the main compound	Low *p*max with high GBDI indicates broad branching; high *p*max with low GBDI indicates canalization
*P*max	Dominance of the main pathway	Distinguishes route-level canalization from distributed pathway investment

**Table 9:** Conceptual delimitation of GBDI relative to established diversity metrics.

Index	Main Input Structure	Primary Question Answered	Why It Is not Equivalent to GBDI
RaoQ	Abundances plus pairwise dissimilarity matrix	How dissimilar are two randomly drawn components?	Depends on an external distance matrix rather than explicit biosynthetic allocation
Functional Hill numbers	Abundances plus functional or trait similarity	How many effectively distinct components exist in a trait-defined space?	Measures effective functional variety, not hierarchical pathway deployment
Bray-Curtis	Two abundance vectors	How compositionally different are two samples?	Between-sample beta-diversity metric, whereas GBDI is a within-sample architectural descriptor
GBDI	Abundances plus pathway attribution	How is chemical abundance distributed across routes and branched within routes?	Designed specifically to describe biosynthetic architecture

**Table 10:** Recommended α-grid for sensitivity analysis of GBDI.

α Value	Expected Behavior	Recommended Use
0.25	Strongly up-weights minor compounds and rare branches	Tests whether conclusions depend on trace-level diversification
0.50	Balanced concave weighting	Primary proof-of-concept value
0.75	Intermediate sensitivity to dominance	Evaluates robustness under weaker rare-compound weighting
1.00	Linear pre-logarithmic contribution	Approaches dominance-weighted pathway investment

## Data Availability

The data supporting the findings of this study are available within the article and its Supplementary Materials. The empirical datasets used for the proof-of-concept application were derived from previously published essential oil composition data cited in the manuscript. The spreadsheet used to calculate GBDI and its companion analytical descriptors is provided as Supplementary File S1.

## Supplementary Materials

The following supporting information can be downloaded at: https://www.mdpi.com/article/10.3390/molecules31122188/s1, Supplementary File: Automated spreadsheet for calculating General Biosynthetic Diversity Index (GBDI) and companion analytical descriptors, hypothetical mixture scenarios used to evaluate the theoretical behavior of the GBDI, pathway-attributed abundance matrices used for GBDI calculation, route-level contributions, between-pathway diversity, intrapathway diversity, dominance metrics, correlation diagnostics with classical descriptors, null-model summaries, and α-sensitivity outputs; Supplementary Note S1. Alpha sensitivity analysis of GBDI; Supplementary Table S1. Interpretation of α values used in the GBDI sensitivity analysis; Supplementary Table S2. Organ-level GBDI sensitivity across α values; Supplementary Table S3. Ontogenetic GBDI sensitivity across α values; Supplementary Table S4. Rank concordance relative to α = 0.50; Supplementary Note S2. Empirical comparison framework with RaoQ, functional Hill numbers, and Bray-Curtis dissimilarity; Supplementary Table S5. Minimum descriptor set for empirical comparison between GBDI and established diversity metrics; Supplementary Table S6. Diagnostic modules recommended for testing complementarity and potential redundancy of GBDI; Supplementary Table S7. Descriptive correlation diagnostics between GBDI and available comparator descriptors; Supplementary Table S8. Interpretation of possible empirical relationships between GBDI and comparator metrics; Supplementary Note S3. Null-model and simulation procedures for testing whether GBDI departs from classical descriptors; Supplementary Table S9. Recommended null-model and simulation procedures for future empirical applications.

## Abbreviations

The following abbreviations are used in this manuscript:

GBDI	General Biosynthetic Diversity Index
*H*^′^	Shannon diversity index
*P*max	Maximum pathway-level dominance
*p*max	Maximum compound-level dominance
Hpath	Between-pathway Shannon diversity
Hwithin,k	Intrapathway Shannon diversity within route k
^1^Dpath	Effective number of biosynthetic routes
^1^Dwithin,k	Effective number of metabolites within route k
GC-MS	Gas chromatography–mass spectrometry
LC-MS/MS	Liquid chromatography–tandem mass spectrometry
PCA	Principal component analysis
R^2^	Coefficient of determination
α	Scaling parameter of the General Biosynthetic Diversity Index
