# Supplementary material for: Beyond Chemodiversity: A General Biosynthetic Diversity Index for Plant Metabolic Architecture in Ecology, Evolutionary, and Bioprospection
Source: Molecules. 2026 Jun 22;31(12):2188. doi: 10.3390/molecules31122188 (PMC13304874; doi:10.3390/molecules31122188)
Supplement: Supplementary file 1 [file molecules-31-02188-s001.zip › Supplementary-Material.pdf]

Supplementary Note S1. Alpha sensitivity analysis of GBDI

The parameter α controls how the pre-logarithmic GBDI aggregate responds to the joint term p_iP_k. Lower α values amplify the relative contribution of rare or minor compounds and minor biosynthetic branches, whereas higher α values progressively emphasize dominant compounds and dominant pathways. The sensitivity analysis was therefore performed to determine whether the empirical interpretation of GBDI depended on a single parameter value.

The grid $\alpha \in \{0.25, 0.50, 0.75, 1.00\}$ was selected because it spans four interpretable regimes: strong rare-branch weighting, balanced concave weighting, intermediate dominance sensitivity, and linear dominance-weighted pathway investment. The same pathway-attributed abundance matrix was used for all α values. For each sample, GBDI values were recalculated, samples were ranked, and rank concordance with the $\alpha = 0.50$ result was evaluated.

Supplementary Table S1. Interpretation of α values used in the GBDI sensitivity analysis

Alpha value	Expected behavior	Use in interpretation
0.25	Strongly up-weights rare compounds and minor branches	Tests whether conclusions depend on trace-level diversification
0.50	Balanced concave weighting	Primary value used in the manuscript
0.75	Intermediate sensitivity to dominance	Evaluates robustness under weaker rare-compound weighting
1.00	Linear pre-log contribution	Approaches dominance-weighted pathway investment

Supplementary Table S2. Organ-level GBDI sensitivity across α values

Organ	GBDI 0.25	Rank	GBDI 0.50	Rank	GBDI 0.75	Rank	GBDI 1.00	Rank
Roots	2.190	2	1.078	3	0.615	4	0.447	3
Stems	1.174	4	0.879	4	0.734	3	0.629	2
Branches	1.958	3	1.192	2	0.831	1	0.650	1
Leaves	2.559	1	1.491	1	0.805	2	0.439	4

Interpretation: organ-level rankings were stable for leaves as the highest-scoring organ under $\alpha = 0.25$ and $\alpha = 0.50$. At higher α values, the ranking shifted toward organs with stronger pathway dominance, particularly branches and stems. This confirms that $\alpha = 1.00$ should be interpreted as a dominance-weighted endpoint rather than as the primary biosynthetic diversity setting.

Supplementary Table S3. Ontogenetic GBDI sensitivity across α values

Phase	GBDI 0.25	Rank	GBDI 0.50	Rank	GBDI 0.75	Rank	GBDI 1.00	Rank
Phase I	1.690	5	0.965	5	0.665	4	0.532	2
Phase II	1.907	4	1.044	4	0.623	5	0.420	4
Phase III	2.240	3	1.295	3	0.697	3	0.361	5
Phase IV	2.400	2	1.415	2	0.899	1	0.622	1
Phase V	2.586	1	1.501	1	0.809	2	0.440	3

Interpretation: the ontogenetic ordering was identical between α = 0.25 and α = 0.50, preserving the developmental pattern Phase V > Phase IV > Phase III > Phase II > Phase I. At α = 0.75, the latest phases remained highest, but Phase IV exceeded Phase V. At α = 1.00, dominance effects became stronger and the ordering changed substantially.

Supplementary Table S4. Rank concordance relative to α = 0.50

Dataset	α compared with 0.50	Spearman ρ	Kendall τ
Organs	0.25	0.80	0.67
Organs	0.50	1.00	1.00
Organs	0.75	0.60	0.33
Organs	1.00	-0.40	-0.33
Ontogenetic phases	0.25	1.00	1.00
Ontogenetic phases	0.50	1.00	1.00
Ontogenetic phases	0.75	0.80	0.60
Ontogenetic phases	1.00	0.10	0.00

Interpretation: high rank concordance at α = 0.25 supports the stability of the primary concave-weighting interpretation. Reduced concordance at α = 0.75 and α = 1.00 indicates a transition from diversification-sensitive behavior to dominance-sensitive behavior.

Supplementary Note S2. Empirical comparison framework with RaoQ, functional Hill numbers, and Bray-Curtis dissimilarity

This supplementary note addresses the reviewer request for a quantitative framework comparing the General Biosynthetic Diversity Index (GBDI) with established diversity and dissimilarity descriptors. The objective is not to claim universal orthogonality or universal non-redundancy, but to provide a reproducible diagnostic structure for evaluating whether GBDI carries information that is not fully explained by classical abundance-based, functional, or beta-diversity metrics in a given dataset.

All descriptors should be calculated from the same normalized sample-by-compound abundance matrix. For GBDI, each compound must additionally be assigned to a primary biosynthetic route. For Rao's quadratic entropy and functional Hill descriptors, a pairwise chemical, structural, biosynthetic, or functional dissimilarity matrix among compounds must be specified and reported. For Bray-Curtis dissimilarity, pairwise sample-level abundance vectors are used. Because the present application is a proof of concept based on mean profiles and a limited number of organ-level and ontogenetic observations, the tests described below are interpreted descriptively and should not be presented as definitive evidence of universal independence among metrics.

RaoQ was defined as the double summation, over all metabolite pairs i and j, of p_i × p_j × d_ij, where p_i and p_j are normalized metabolite abundances and d_ij is the selected pairwise dissimilarity between metabolites i and j. Bray-Curtis dissimilarity between samples a and b was defined as the sum of |p_ai - p_bi| divided by the sum of (p_ai + p_bi) across all metabolites. Functional Hill descriptors should be computed from the same abundance matrix and a declared trait or similarity structure, using a stated diversity order q. These choices are not neutral; therefore, the distance matrix, trait coding, transformation, and value of q must be reported together with the results.

Supplementary Table S5. Minimum descriptor set for empirical comparison between GBDI and established diversity metrics.

Descriptor	Required input	Primary analytical target	Use in relation to GBDI
GBDI	Abundance matrix plus biosynthetic-route labels	Within-sample biosynthetic architecture	Integrates route allocation with intrapathway diversification; not a pairwise distance metric.
Shannon H', Simpson 1-D, Pielou J, richness	Abundance matrix	Compound-level alpha diversity, dominance, and evenness	Baseline descriptors; they do not encode biosynthetic route attribution.
RaoQ	Abundance matrix plus compound-by-compound dissimilarity matrix d_ij	Abundance-weighted expected dissimilarity among metabolites	Tests whether GBDI parallels chemical, structural, functional, or biosynthetic dissimilarity, depending on d_ij.
Functional Hill numbers	Abundance matrix plus functional/trait similarity or dissimilarity matrix and diversity order q	Effective number of functionally distinct compounds	Tests whether GBDI tracks functional variety or reflects pathway deployment.
Bray-Curtis dissimilarity	Pairs of normalized sample abundance vectors	Between-sample compositional turnover	Evaluates whether samples that differ in composition also differ in biosynthetic architecture.
Hpath, Dpath, Hwithin,k, pmax, Pmax	Pathway-attributed abundance matrix	Route allocation, intraroute branching, and canalization	Diagnostic companion descriptors used to interpret why GBDI increases or decreases.

The empirical comparison should be performed in sequential modules. First, calculate compound-level alpha descriptors and dominance measures. Second, calculate GBDI and its pathway-level decomposition. Third, calculate RaoQ and functional Hill descriptors under explicitly stated dissimilarity or trait matrices. Fourth, evaluate Bray-Curtis dissimilarity on compound-level abundance vectors and, when relevant, on pathway-aggregated abundance vectors. Finally, compare these outputs using correlation diagnostics, rank concordance, ordination, variance partitioning, and null-model or simulation procedures.

Supplementary Table S6. Diagnostic modules recommended for testing complementarity and potential redundancy of GBDI.

Module	Comparison structure	Recommended output	Interpretation
Correlation screen	GBDI versus Shannon, Simpson, Pielou, richness, RaoQ, functional Hill numbers, Hpath, pmax, and Pmax	Pearson r, Spearman rho, Kendall tau	High correlation suggests shared information; low or moderate correlation supports complementarity, but not universal independence.
Rank concordance	Sample ranks generated by GBDI and comparator metrics	Spearman rho and Kendall tau	Tests whether GBDI changes sample prioritization relative to classical descriptors.
Multivariate comparison	Bray-Curtis matrices, pathway-aggregated matrices, and route-contribution matrices	PCoA/NMDS, Procrustes comparison, Mantel test when justified	Evaluates whether compound turnover and pathway architecture occupy similar multivariate spaces.
Variance partitioning	GBDI as response or explanatory variable; classical alpha metrics, dominance, and pathway descriptors as predictor blocks	Adjusted R2, partial R2, redundancy analysis, or distance-based RDA	Quantifies the proportion of GBDI-associated variance explained by compositional, dominance, and pathway components.
Simulation/null models	Route-label permutations, within-route abundance permutations, and abundance resampling	Observed value, null mean, null SD, z-score, empirical p value, and rank stability	Tests whether observed GBDI is greater or lower than expected under random route attribution or random allocation.

The following values illustrate the minimum empirical diagnostic recommended by the reviewer. They were calculated from the summarized GBDI and classical descriptors reported for the organ-level and ontogenetic sections of the revised manuscript. Because n = 4 for organs and n = 5 for ontogenetic phases, these coefficients are descriptive and are not used to claim statistical independence. The full compound-level matrix is still required for definitive RaoQ, functional Hill, Bray-Curtis, and variance-partitioning analyses.

Supplementary Table S7. Descriptive correlation diagnostics between GBDI and available comparator descriptors.

Dataset	Comparison	Pearson r	Spearman rho	Kendall tau	Interpretation
Organ-level dataset (n = 4)	GBDI versus Shannon H'	0.604	0.400	0.333	Moderate association only; organ-level GBDI is not a simple recoding of Shannon diversity.
Ontogenetic phases (n = 5)	GBDI versus developmental order	0.972	1.000	1.000	Strong directional association; interpreted descriptively because only five ordered phases are available.
Ontogenetic phases (n = 5)	GBDI versus Shannon H'	0.868	0.700	0.600	Shared trend with incomplete rank agreement; Phase IV illustrates divergence between Shannon diversity and biosynthetic architecture.
Ontogenetic phases (n = 5)	GBDI versus Pielou J	0.730	0.700	0.600	Evenness explains part of the variation but does not exhaust the pathway-informed interpretation.

These diagnostics support a cautious interpretation. GBDI may covary with compound-level diversity when higher richness and evenness are accompanied by broader pathway allocation or intrapathway branching. However, partial rank disagreement indicates that GBDI should not be reduced to Shannon diversity, Pielou evenness, or developmental ordering. This is especially important in the Phase IV comparison, where Shannon diversity decreases relative to Phase III while GBDI continues to increase, indicating that a profile may become more compositionally dominated while retaining or increasing pathway-informed organization.

Supplementary Table S8. Interpretation of possible empirical relationships between GBDI and comparator metrics.

Observed relationship	Likely analytical meaning	Recommended interpretation
High GBDI-Shannon correlation and high GBDI-Hpath correlation	Compound richness/evenness and route allocation increase together.	Report GBDI as concordant with classical diversity and avoid claims of independent information.
Moderate GBDI-Shannon correlation but high GBDI-Hwithin,k	A dominant route is internally branched even if compound-level alpha diversity is not maximal.	Interpret as focused but branched metabolism.
High Shannon diversity but low GBDI	Many compounds are present but concentrated within weakly expressed or canalized route structures.	Do not interpret richness alone as biosynthetic architectural expansion.
High Bray-Curtis turnover but similar GBDI	Samples differ in compound identity but preserve similar route-level organization.	Treat compositional turnover and biosynthetic architecture as complementary dimensions.

Observed relationship	Likely analytical meaning	Recommended interpretation
Low Bray-Curtis turnover but different GBDI	Compound profiles are similar in abundance space, but route allocation or intrapathway branching differs.	Investigate pathway assignment, route contributions, and dominance descriptors.
RaoQ/functional Hill high but GBDI low	Metabolites occupy broad structural or functional space but are not broadly deployed across biosynthetic architecture.	Interpret as structural heterogeneity without necessarily broad pathway deployment.

Supplementary Note S3. Null-model and simulation procedures for testing whether GBDI departs from classical descriptors

A null-model layer is recommended to strengthen empirical claims regarding complementarity. The route-label permutation null model randomly reassigns biosynthetic labels among compounds while preserving the abundance vector and, when desired, the number of compounds per route. This tests whether the observed GBDI depends on the empirical organization of compounds into biosynthetic routes. A within-route abundance permutation null model preserves route membership but randomizes abundance allocation within each route, testing whether intrapathway dominance drives the observed score. A Dirichlet or bootstrap resampling model can be used to propagate uncertainty in relative abundance estimates when replicating or peak-area uncertainty is available.

For each null model, the recommended output is the observed GBDI, the mean and standard deviation of the null distribution, the standardized effect size z = (observed GBDI - null mean) / null SD, an empirical two-sided p value, and the stability of sample ranks across iterations. The same procedure can be applied to RaoQ and functional Hill numbers by recalculating them under alternative dissimilarity matrices, such as structural fingerprints, biosynthetic-class distances, or hybrid chemical-biosynthetic distances. This approach directly addresses whether apparent complementarity is robust to reasonable analytical choices.

Supplementary Table S9. Recommended null-model and simulation procedures for future empirical applications.

Procedure	Constraint preserved	Question addressed	Minimum output
Route-label permutation	Abundance vector preserved; route labels randomized	Tests whether observed GBDI depends on empirical biosynthetic attribution.	Observed GBDI, null mean, SD, z-score, empirical p value, rank stability.
Within-route abundance permutation	Route membership preserved; abundances randomized within each route	Tests whether intrapathway dominance or branching drives GBDI.	Same as above, plus route-level contribution stability.
Abundance resampling	Relative abundances resampled by bootstrap or Dirichlet model	Propagates analytical uncertainty in peak areas or relative abundance estimates.	Confidence interval for GBDI and comparator metrics.
Alternative distance matrices for RaoQ and functional Hill numbers	Structural, biosynthetic, functional, and hybrid dissimilarity matrices	Tests whether conclusions depend on how functional or chemical distance is encoded.	Sensitivity of RaoQ, functional Hill numbers, and their correlations with GBDI.
Bray-Curtis on compound and pathway matrices	Dissimilarity calculated on compound-level and pathway-aggregated abundance vectors	Separates compound turnover from pathway-level turnover.	Ordination plots, pairwise matrix correlation, and Procrustes or Mantel statistics when sample size allows.

Taken together, these supplementary procedures convert the comparison with RaoQ, functional Hill numbers, and Bray-Curtis dissimilarity from a purely conceptual discussion into a testable analytical workflow. The appropriate conclusion for the present proof-of-concept dataset is that GBDI is complementary and dataset-dependent. Stronger claims of non-redundancy, orthogonality, or independence should only be made when supported by expanded datasets, explicit distance matrices, variance partitioning, and null-model validation.
